# Enhancing career adaptability in college students: a Tai Chi-based sports intervention study

**DOI:** 10.3389/fpsyg.2024.1455877

**Published:** 2024-09-26

**Authors:** Le Wang, Yuanyan Zhai, Qichao Sun

**Affiliations:** ^1^Department of Physical Education, Shandong University of Traditional Chinese Medicine, Jinan, China; ^2^College of Health Sciences, Shandong University of Traditional Chinese Medicine, Jinan, China; ^3^Guangdong Justice Police Vocational College, Guangzhou, China

**Keywords:** career adaptability, Tai Chi sports, basic psychosocial needs, sports intervention, college student

## Abstract

**Introduction:**

This research explores the impact of Tai Chi, a traditional Chinese martial art, on the career adaptability of college students, utilizing a quasi-experimental design. With the increasing complexities in the transition from school to work, effective interventions that address both psychological and vocational needs are essential.

**Methods:**

The study involved 70 senior college students, randomly assigned to either an intervention group that participated in an organized 8-week Tai Chi program based on positive youth development (PYD) or a control group with no intervention.

**Results:**

Results indicated significant improvements in the intervention group in terms of both career adaptability and satisfaction of basic psychological needs, compared to the control group. Mediation analysis revealed that the increase in career adaptability induced by intervention was mediated by the satisfaction of basic psychological needs, underscoring the effectiveness of Tai Chi as a holistic intervention tool.

**Discussion:**

This study contributes to the field by demonstrating that physical activity, particularly one embedded with deep cultural and philosophical significance like Tai Chi, can effectively enhance the career adaptability of college students. It advocates for the inclusion of PYD-based physical practices in developmental interventions aimed at preparing youth for the challenges of the modern workforce.

## 1 Introduction

The transition from college to the professional world presents significant challenges for college students, particularly against the backdrop of unfavorable employment conditions and rising unemployment rates, which have captured the attention of global governmental and non-governmental organizations (van der Horst et al., 2021). The concept of individuals’ relative plasticity, which refers to the potential for systematic changes throughout their lifespan (Savickas, 2002), has inspired researchers to explore a range of interventions that foster proactive development within individuals and their contexts.

Career adaptability is defined as “the readiness to cope with the predictable tasks of preparing for and participating in the work role and with the unpredictable adjustments prompted by changes in work and working conditions” (Savickas, 1997, p. 254). It includes career concern (prepare and plan for career development), career control (responsibility and control over one’s career), career curiosity (exploration of self-learning and advancement opportunities), and career confidence (belief in one’s problem-solving abilities; Savickas and Porfeli, 2012). Career adaptability is increasingly recognized as a critical socio-psychological resource for successful environmental adaptation and is highly valued by employers (Guan et al., 2016; Hirschi and Valero, 2015). Research in this area often focuses on the impact of family members’ interaction, individual traits, and workplace factors on career adaptability (e.g., Lu and Jia, 2022; Xia et al., 2020). For instance, Guan et al. (2015) found that career-specific parental behaviors (including lack of engagement, parent support, and intervention) have different impacts on the career adaptability of college students. Specifically, parental engagement and support plays a positive role, while excessive intervention can have negative effects. Besides, neuroticism has been found to negatively predict career adaptability among college students (Zacher, 2014), whereas transformational leadership positively affects it (Wang et al., 2017).

Career adaptability is a learnable skill that can be enhanced through education and experience (Savickas, 2005), making the exploration of scientifically effective intervention methods a significant direction in this field. Currently, interventions of career adaptability primarily target college students who are at the formative stage of their career, making interventions potentially more socially meaningful (e.g., Koen et al., 2012). This means that intervention at this stage may yield longer-term benefits for individuals, organizations and society. Traditionally, career intervention research has concentrated on knowledge-based vocational training, such as career information training (e.g., Janeiro et al., 2014; Liu et al., 2023), and one-on-one counseling (e.g., Obi, 2015). Given the importance of career interventions for college students, researchers have begun to explore how other context-based intervention approaches, such as internships (Ocampo et al., 2020), can influence their career adaptability. It is necessary for researchers to further explore the expansion of intervention paradigms.

The interplay between physical and psychological factors suggests that physical activity interventions could effectively enhance career adaptability. From the perspective of positive youth development program (PYD), sports intervention programs can foster beneficial interactions between social backgrounds and individual growth (Larson, 2000). PYD programs assert that positive development stems from synergistic relationships within ecological settings (Lerner et al., 2009, 2011). Structured sports intervention programs could provide rich interactional contexts and create positive and safe environments for participants (Bruner et al., 2023). Multiple qualitative studies show that PYD-based sports interventions promote positive self-perceptions and help participants understand themselves (Camiré et al., 2009), as participants in Armour and Sandford (2013) said, participating in sports training programs made them “braver and more confident,” which in line with the implication of career confidence dimension.

According to trait activation theory, environmental cues related to traits—including task, social, and organizational cues—trigger the expression of individual traits (Tett and Burnett, 2003; Tett and Guterman, 2000). The adaptation process is a continually mutual adjustment between individuals and their environments (Savickas, 2013). Structured sports programs, replete with such environmental cues, can boost participants’ initiative and intrinsic motivation, thereby promoting career development (Barton, 2011; Larson, 2000).

Research supports the positive impact of PYD-based sports programs on one’s development across various domains. The structured elements of sports environments, including sport tasks *per se*, sports characteristics (like rules, decision-making, relationships, and competition), and key environmental elements (like coaches and peers) significantly contribute to positive youth development (Camiré et al., 2011; Johnston et al., 2013). Studies have also revealed the important role of sports training programs in promoting individual positive adaptation and self-regulation (e.g., Moran et al., 2019; Mosoi et al., 2020), suggesting that sports could also be a potential way to effectively prepare individuals for work roles. However, empirical evidence is still lacking on whether and how this type of intervention promotes the development of individual career adaptability, highlighting a gap in the theoretical and practical frameworks of career development interventions.

Trait activation theory elucidates how situational factors can elicit related traits, yet it does not fully address the psychological mechanisms behind this interaction. Intervention theories grounded in positive development suggest that when intervention programs provide a need-supportive motivational climate, participants can experience psychological need satisfaction, leading to optimal outcomes of psychological development (Gould and Carson, 2008). Evidence from empirical studies also showed that the group structure of sports training provided a platform for individual interaction (e.g., communication) and the sharing of group states (e.g., cohesion), influencing individuals’ intrinsic motivation (Riemer and Chelladurai, 1998; Smith and Smoll, 2002). Furthermore, coaches play a critical role in this group structure, and when coaches exhibit more autonomy-supportive behaviors, athletes experience the satisfaction of autonomous needs, enhancing their intrinsic motivation (Gagné et al., 2003). The other study employed handball training revealed that when coaches allow a high level of participatory atmosphere, participants showed higher intrinsic motivation and commitment (Alesi et al., 2019).

This study aims to design an intervention program based on the positive youth development perspective and trait activation theory. The first objective is to explore the impact of sports interventions on college students’ career adaptability. The second objective is to update traditional coaching methods by incorporating features of positive youth development in the intervention program. This includes guiding and encouraging a motivational social and activity environment, such as supporting team members’ agency, as suggested by Larson et al. (2019). Furthermore, the study aims to investigate the motivational mechanisms that contribute to participants’ career development through sports training. Specifically, we hypothesize that a structured Tai Chi training program can lead to positive development outcomes by satisfying individuals’ basic psychological needs. By doing so, our study aims to enhance the findings and theoretical framework of interventions targeting college students’ career adaptability.

### 1.1 The design of Tai Chi intervention program

Tai Chi, a traditional Chinese martial art, embodies profound philosophical principles that set it apart from other forms of exercise. Central to its practice is the principle of Yin and Yang, positing that all elements exist in a mutually dependent yet opposing relationship (Wayne and Kaptchuk, 2008). This practice emphasizes enhancing awareness and cultivating sensitivity to one’s body and external environment. Research indicates that this introspective ability helps individuals better adapt to changes and stresses in life, and cope with challenges and difficulties more effectively (Wang et al., 2014). As participants gradually internalize the values promoted by the intervention training, their behaviors are more likely to be self-regulated (Gould and Carson, 2008).

The study proposes a systematical Tai Chi intervention program that integrates dimensions of physical, cognitive, and interpersonal interactions. This approach aims to enable participants to think and act adaptively when facing career-related challenges. Coaches play a pivotal role in this context, significantly influencing the efficacy of the interventions (Ferrari et al., 2017; Rathwell et al., 2020). Therefore, this study will incorporate techniques from PYD-based sports programs, focusing on creating a motivating and safe atmosphere (Rodrigues et al., 2020); establishing goal-oriented practices with clear, consistent rules and expectations (Côté et al., 2007); and fostering autonomy and participation by such as involving team members in the design and execution of training strategies and goals (Larson et al., 2019). Additionally, the intervention emphasizes cultivating positive social interactions between coaches and team members, characterized by supportive relationships and effective communication (Strachan et al., 2011). This structured approach is designed to explore and enhance the motivational pathways for participants’ positive development, aiming to improve their adaptability in their career trajectories.

## 2 Hypothesis development

### 2.1 The influence of sports training on undergraduates’ career adaptability

Career adaptability is a socially constructed process (Savickas, 2013). Based on trait activation theory, the context of sports training may activate individuals’ adaptive traits.

Within the framework of sports activities, participants typically engage under the guidance of their coaches, who serve as significant environmental cues. Coaches can positively impact participants’ understanding of their career interests, skills, and values, facilitating better coping mechanisms for challenges and changes, and fostering strong adaptability (Rasberry et al., 2011; Vella et al., 2011). Moreover, the sports environment cues such as rules, expectations, and responsibilities outlined in sports interventions (Perkins and Noam, 2007), and competitive tasks (e.g., training and competitions) have been shown to support participants’ social adaptation (Eime et al., 2013; Mosoi et al., 2020). For instance, a study on a winter sports intervention aimed at enhancing college students’ social adaptability found that physical exercise helps maintain composure and effectively cope with competitive pressures, thus bolstering social adaptability (Zhang and Xia, 2021). Additionally, research on young elite athletes indicated that the tasks emphasized in sports training—planning, exploration, and decision-making—are closely linked to career adaptability development (Ojala et al., 2023). The sports environment, by mimicking career-related tasks such as problem-solving and attention regulation, may significantly contribute to the development of young individuals’ career planning and control.

Therefore, it is hypothesized that participation in sports training positively influences the career adaptability of college students.

*Hypothesis 1*: Compared to the control group, the career adaptability of participants in intervention group will be significantly improved following Tai Chi training.

### 2.2 The mediating role of basic psychological needs

Basic psychological needs, which include autonomy, competence, and relatedness, are fundamental for human motivation and well-being (Deci and Ryan, 2000). Autonomy involves the experience of volition and choice; competence relates to the feelings of self-efficacy in the environment; and relatedness pertains to the sense of belongingness and connection with others in the social context (Ryan and Deci, 2000). Research suggests that there are three environmental factors that facilitate the satisfaction of individual psychological needs: (a) autonomy support, (b) structure, and (c) involvement (e.g., Connell and Wellborn, 1991; Reeve et al., 1999). Autonomy support involves providing choices and opportunities (e.g., Ryan and Deci, 2000); structure relates to competence needs as it involves establishing clear expectations, confidence in task success, and providing constructive feedback (Markland et al., 2005); involvement, pertaining to relatedness, reflects the degree to which individuals perceive significant others as genuinely interested in their welfare (Connell and Wellborn, 1991).

The organized sports environment, exemplified by disciplines such as Tai Chi, embodies these elements. Tai Chi environment is goal-oriented, emphasizing comprehension of rules and expectations. Coaches, driven by the requirements of their job and Tai Chi principles, need to listen to participants, and providing feedback and adjust training strategies accordingly, thereby enhancing goal orientation, facilitating successful experiences, and fostering connections (Fenton et al., 2014; Solstad et al., 2015). Research suggests that such sports training environments boost self-efficacy and interpersonal skills, thereby satisfying basic psychological needs (Rodrigues et al., 2020). Therefore, we hypothesize:

*Hypothesis 2*: Compared to the control group, Tai Chi training in the intervention group promotes the satisfaction of basic psychological needs.

The satisfaction of basic psychological needs equips individuals with substantial psychological resources, enabling better coping with challenges and adaptability in uncertain work conditions (Deci and Ryan, 1985; Olafsen and Deci, 2020). This satisfaction is positively linked to effective career decision-making and goal-setting in college students (Holding et al., 2020; Pesch et al., 2018). Studies show that the extent to which an individual’s basic psychological needs are met is crucial for their career adaptability (Şahin and Gülşen, 2022), promoting quick reaction to workplace uncertainties (Collie and Martin, 2017), and encouraging participation in career development activities (Guan et al., 2016). Career adaptability entails the readiness to explore the work environment and prepare for unfamiliar situations, suggesting that satisfying basic psychological needs fosters this readiness.

Career adaptability could be acquired from education and life experiences, and it is based on relationships and social construction (Hirschi and Valero, 2015). Individuals naturally pursue opportunities to meet their own needs, and these needs and expectations shape their participation in the environment (Skinner et al., 2014). Therefore, providing an educational environment that can meet basic psychological needs may be an important way to improve individual career adaptability. In general, we speculate that the PYD-based Tai Chi exercise training can shape a positive development environment in terms of autonomy, structure, and participation, in order to meet the basic psychological needs of team members, thereby promoting the career adaptability. Therefore, we hypothesize that:

*Hypothesis 3*: The satisfaction of basic psychological needs mediates the relationship between Tai Chi training and career adaptability in college students.

## 3 Methods

### 3.1 Subjects

Seventy senior college students were recruited to participate in this intervention study from compulsory courses at a university of China. They were randomly assigned to two groups: the intervention group and the control group, with 35 participants each. The intervention group consisted of 45.7% males, and the control group consisted of 51.4% males. The average age of participants was 20.9 years. Before the experiment began, all participants were informed of the voluntary nature of their participation and signed an informed consent form. This study has been reviewed and approved by the Ethics Committee of Shandong University of Traditional Chinese Medicine.

### 3.2 Experimental design and procedures

The study utilized a 2 (between-subjects factor: intervention group, control group) × 2 (within-subjects factor: pretest, posttest) mixed experimental design. The primary analytical methods included t-tests, covariance analysis, and regression analysis to evaluate the main effects of the Tai Chi training intervention on career adaptability and explore the mediating mechanisms involved.

The intervention group participated in an 8-week Tai Chi training program led by a professional coach. The control group did not receive any form of intervention during this period. The course was conducted once a week for 45 min each time. To ensure that the PYD and psychological techniques required for the intervention were correctly implemented, the coach was trained by two doctoral students specializing in the field of psychological counseling during the first 3 weeks of the intervention.

The psychological techniques taught to coaches primarily include: (1) utilizing expressions that fully convey empathy, complimenting, and cheerleading (e.g., “You are doing great,” “You are learning quickly,” “I have encountered similar technical challenges before, and I found that adjusting my mindset helped significantly. Would you like to give it a try?”). (2) Listening techniques, such as open body posture, maintaining eye contact, refraining from jumping to conclusions or labeling. (3) Unconditional positive regard. We required the coach to maintain unconditional positive regard for the players and encourage the same among team members. (4) Self-suggestion training is also incorporated, guiding players to use effective self-suggestion like, “It’s okay to make mistakes; I can seize the next opportunity.” (5) Cognitive restructuring. It involves identifying irrational beliefs, such as “I must win, or it is meaningless,” and replacing them with more constructive thoughts, like “What I need to focus on is doing my best and controlling each movement carefully.”

During intervention period, coaches are required to set clear goals and provide feedback at any time. They are also encouraged to avoid using disparaging or negative language, instead adopting encouraging and respectful words and attitudes. A doctoral student accompanies each training session to provide supervision and guidance.

Unlike traditional training sessions, each session in this program consists of four components: relaxation exercises, skill training, competition simulation, and sharing and discussion. During each sharing and discussion session, players are encouraged to share with each other any insights and feelings from the training session that have contributed to their personal development. Coaches are required to apply listening techniques and unconditional positive regard, using encouraging language to create an open and equal atmosphere.

Measurements of career adaptability and basic psychological needs were conducted for both groups 1 week before and 1 week after the intervention. During the pretest, participants also provided basic demographic information, including gender and age. All measurement questionnaires were distributed to participants through an online platform.

### 3.3 Measures

Career adaptability. This variable is measured using a scale developed by Savickas and Porfeli (2012) with a total of 24 items. We use the Chinese version translated by Hou et al. (2012), which has been used in previous local studies (such as Guan et al., 2016). Example items include “I will observe different ways of doing things.” The scale uses a Likert 5-point scale, and participants need to evaluate each item from 1 to 5 (1 = strongly disagree to 5 = strongly agree) based on their actual situation. The Cronbach α value of this scale is 0.82.

Basic psychological needs. The basic psychological needs scale developed by Johnston and Finney (2010) was used for evaluation, which included 21 items such as “I generally feel free to express my ideas and opinions,” “I often do not feel very capable,” and “People in my life care about me.” The scale uses a 7-point Likert scale, and participants are required to evaluate each item from 1 to 7 based on their actual situation (1 = strongly disagree, 7 = strongly agree). The higher the score, the more satisfied they feel. In the current study, the Cronbach α value is 0.87.

## 4 Results

### 4.1 Main effects of the intervention

To test hypotheses 1 and 2, which evaluate the main effects of Tai Chi intervention on participants’ basic psychological needs and career adaptability, we first conducted independent sample *t*-tests to compare the pretest scores of the intervention and control groups. The results showed no significant differences at the baseline between the two groups in terms of basic psychological needs (*t* = −0.01, *p* > 0.05) and career adaptability (*t* = −0.55, *p* > 0.05), confirming that the initial conditions were comparable.

Following the intervention, posttest comparisons revealed that the intervention group exhibited significantly higher levels of both basic psychological needs (*t* = 2.09, *p* < 0.05) and career adaptability (*t* = 3.56, *p* < 0.05) compared to the control group. Descriptive statistics of pretest and posttest for each group have shown in Table 1. These findings provide preliminary support for the positive effects of the Tai Chi intervention.

**Table 1:** Descriptive statistics of pretest and posttest for each group.

	Pretest		Posttest	
	*SD*	*M*	*SD*	*M*
CA_intervention_	0.62	3.56	0.55	4.04
CA_control_	0.70	3.66	0.65	3.53
BN_intervention_	1.08	5.02	1.09	5.57
BN_control_	1.28	4.97	1.14	4.98

CA, career adaptability; BN, basic psychological needs.

**Figure 1:**
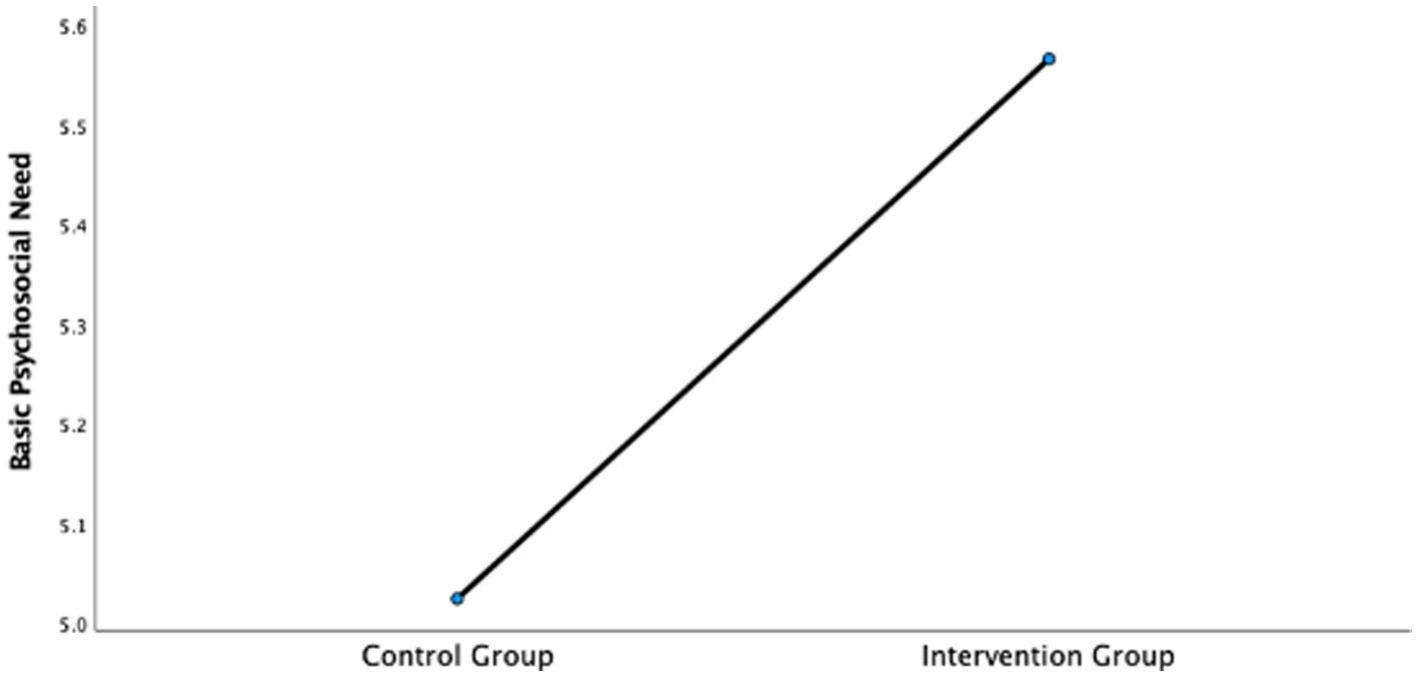
The results of covariance analysis of basic psychological needs.

**Figure 2:**
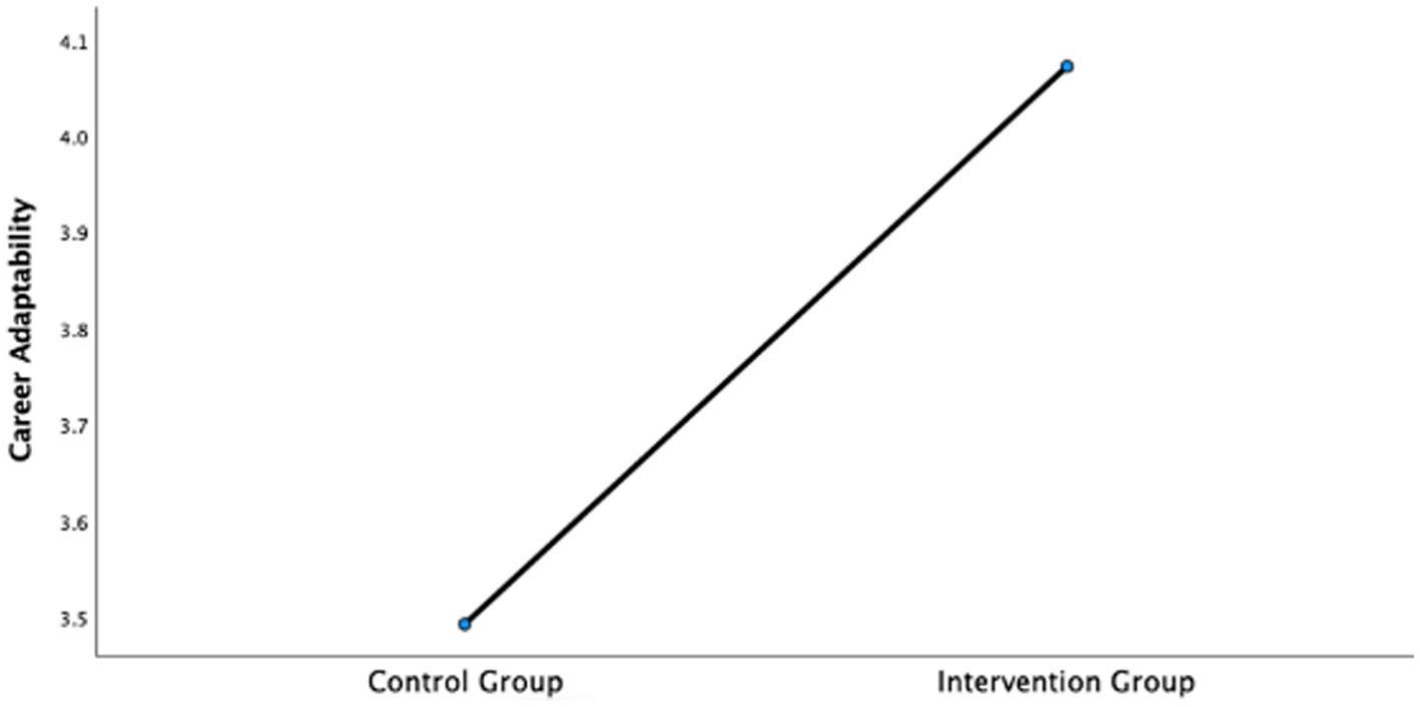
The results of covariance analysis of career adaptability.

To further account for potential confounding variables such as pretest scores, age, and gender, a covariance analysis (ANCOVA) was conducted. Homogeneity of variance tests were conducted first (basic psychological needs: *F* = 2.03, *p* > 0.05; career adaptability: *F* = 1.49, *p* > 0.05), meeting the assumptions for ANCOVA. After controlling for the pretest levels, participants’ age and gender, the results also confirmed the positive impact of the intervention, with significant improvements observed in both basic psychological needs (*F* = 6.24, *p* < 0.05) and career adaptability (*F* = 26.9, *p* < 0.01), supporting Hypotheses 1 and 2 (as shown in Figures 1, 2).

### 4.2 Mediation analysis

**Figure 3:**
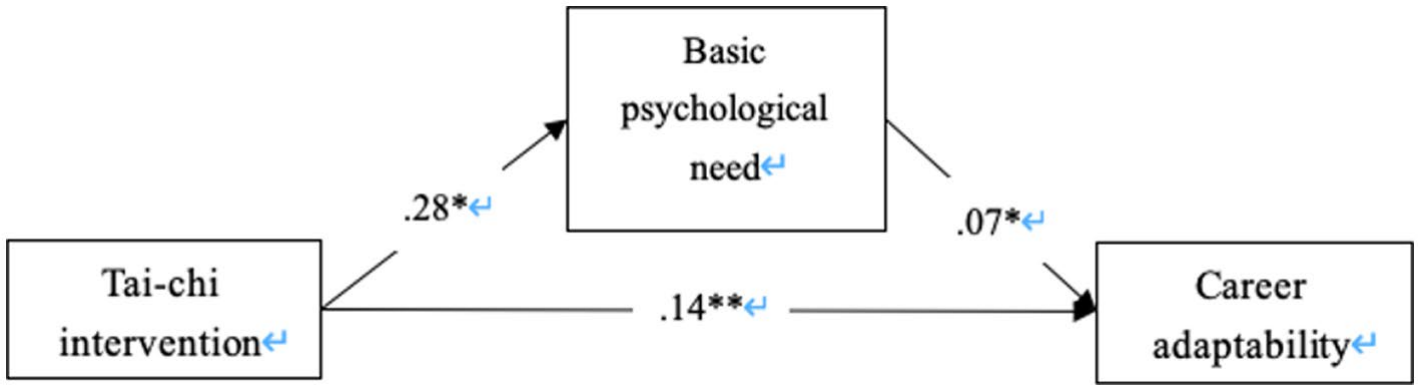
The mediation model. *p < .05, **p < .01.

A mediation analysis was performed to assess whether the enhancement in basic psychological needs could explain the improvement in career adaptability post-intervention. Since the exercise intervention manipulation was a categorical variable, different levels of the manipulation were coded as dummy variables, with the intervention group and control group coded as 1 and 0, respectively. The correlations among the main variables of interest are presented in Table 2, and all variables demonstrated significant correlations in the expected directions. The PROCESS Macro program was utilized to test the mediation effects. The results revealed a significant indirect effect (*B* = 0.04, CI [0.01, 0.11]). Specifically, Tai Chi exercise enhanced participants’ levels of basic psychological needs (*B* = 0.28, CI [0.07, 1.09]), which in turn improved their career adaptability (*B* = 0.14, CI [0.33, 0.74]), as illustrated in Figure 3. Hence, Hypothesis 3 was supported. These findings suggest that Tai Chi not only directly benefits psychological and career-oriented outcomes but also facilitates these benefits through the satisfaction of basic psychological needs.

**Table 2:** The correlation between main variables in this study.

Variable	1	2	3
1. Intervention manipulation			
2. Posttest of basic psychological needs	0.25*		
3. Posttest of career adaptation	0.16**	0.28**	

*p < 0.05, **p < 0.01.

## 5 Discussion

College students, at the stage of emerging adulthood, encounter various developmental pressures including role conflict, career exploration, and the school-to-work transition. Young adults often lack essential information for making informed career decisions, leading to uncertainty and impulsive job choices (Arnett, 2007; Kelly and Lee, 2002). This concern underscores the importance of developing effective theory-based interventions that can guide policy and decision-making (van der Horst et al., 2021). This study initially incorporates traditional Chinese martial arts, Tai Chi, as a method for enhancing career adaptability among college students. This study demonstrates that Tai-chi sports interventions enhance career adaptability among college students by meeting their basic psychological needs. Specifically, participants in the intervention group exhibited significant improvements in both career adaptability and basic psychological needs—enhancements not observed in the control group. These findings demonstrate that even short-term PYD-based Tai Chi training can significantly boost career adaptability, mediated by the fulfillment of basic psychological needs. These results suggest that Tai Chi promotes significant career development.

Coaches play an important role in the PYD-based sports intervention. In this study, coaches are required to encourage, compliment, and provide unconditional positive regard to players, guiding them in using positive self-suggestions and coping with challenges, promoting participants’ career adaptability. After each session, a summary and sharing were conducted, creating a virtuous cycle that led to improvements in participants’ skill levels and enhanced their confidence. Coaches demonstrate full respect for individual decision-making, encouraging active participation, which fosters the development of autonomy. At the same time, players experience a stronger sense of belonging within a motivating and safe interpersonal environment that coaches trying to create. This approach aligns with the 4Cs (competence, confidence, connection, and caring) principles emphasized in the PYD-based intervention framework (Côté et al., 2010), and enhancing the effectiveness of the intervention in this study.

### 5.1 Expanding intervention paradigms

The combined effects of individual characteristics and environmental factors influence and promote the design of interventions for positive development. The current study contributes to a broader understanding of how non-traditional interventions, such as Tai Chi, can be effectively utilized. Sports interventions can strengthen the perception-action cycle of individuals, stimulating cognitive and affective feedback through their physical actions and perceptual experiences (Gibson, 2014). This cyclic process helps individuals better understand their behaviors and reactions, enabling them to adapt to the demands and challenges of the work environment. The integration of PYD sports interventions into vocational psychology represents a novel approach, contributing to the theoretical development of career interventions.

PYD programs focus on optimizing developmental opportunities within specific environments, suggesting that organized activities can significantly enhance developmental experiences (Larson and Tran, 2014). While PYD-based sports interventions have been recognized for their value in promoting individuals’ positive development (e.g., Holt and Neely, 2011), their application in vocational psychology has been limited. This study illustrates how organized sports training programs offer a platform for fostering career adaptability by providing a social environment for college students, including coaches, peers, tasks, and competitive situations. The interaction between individuals and their social environment promotes one’s development, and the potential for behavioral and developmental changes exists within this dynamic interaction (Lerner, 2002).

### 5.2 The mediating role of basic psychological needs

This research not only demonstrates the main effects of Tai Chi on career adaptability but also illuminates the underlying mechanisms through the mediating role of basic psychological needs. This aspect is often overlooked in career intervention study, which may restrict the progress of intervention theories in this field. Satisfaction of psychological needs is influenced by the environment (Ryan and Deci, 2000), and individuals strive for the satisfaction of basic psychological needs, shaping their engagement with the environment (Skinner et al., 2014). This study adds to the knowledge base of this field by providing a motivational perspective.

A critical question in career intervention research is the extent to which intervention effects can generalize to overall career success. Aligning with previous findings, our findings suggest that when individuals’ basic needs are satisfied, intervention outcomes may extend to other domains of life (e.g., Ntoumanis et al., 2021). The satisfaction of these needs is pivotal in translating the values and self-regulatory skills acquired through sports into enhanced career adaptability, offering a promising pathway for future research and practical applications in vocational psychology and beyond.

### 5.3 Limitations and directions for future research

This study has certain limitations that provide directions for future research. Firstly, while this study provided valuable insights into the effects of Tai Chi training on the career adaptability of college students, its focus was limited to this specific demographic. As societal trends shift toward prolonged educational periods and delayed entry into the workforce, the definition of emerging adulthood extends. Future research could beneficially explore the applicability of sports interventions to other groups within this stage, such as young employees who have recently entered the workforce. This would help to understand the broader impacts of sports interventions on career adaptability across different stages of life transitions.

Secondly, the current study primarily examined the motivational pathway of sports training on career adaptability. However, sports activities are also known to influence cognitive functions, such as attention regulation, which may be crucial for career development (Moran and Toner, 2017). Future studies should investigate these cognitive aspects to uncover additional mechanisms through which sports training might enhance career adaptability. This exploration could provide a more comprehensive understanding of how physical activities influence one’s career growth.

Thirdly, Tai Chi, characterized by its integration of physical movement with ethical principles and philosophical elements of traditional Chinese culture, differs significantly from more conventional sports like running or strength training. Therefore, it is necessary to further explore the activation effects and mechanisms of other sports interventions on individuals’ psychological traits. Future intervention research should take into consideration the characteristics of the specific sports activities.

Additionally, the Tai Chi intervention utilized in this study was predominantly an individual-focused activity without competitive elements, particularly in the initial stages of training. In contrast, team sports like soccer or basketball inherently include cooperative dynamics and competitive interactions, which could offer different developmental benefits and challenges. Investigating the impacts of team-based sports on career adaptability could reveal whether the cooperative and competitive elements of these sports provide additional or distinct advantages for career development. Future studies should explore these dynamics to determine if the positive effects of sports on career adaptability observed in Tai Chi training are replicable in team sports settings and to elucidate the underlying mechanisms.

By addressing these limitations and exploring these suggested directions, future research can significantly expand the understanding of how diverse forms of physical activity influence career adaptability and professional development, thereby contributing to more effective and tailored intervention strategies.

## 6 Conclusion

This study employed a quasi-experimental intervention research design to explore the impact of Tai Chi training, informed by the Positive Youth Development (PYD) framework, on enhancing career adaptability among college students. The findings demonstrate that Tai Chi, a martial art deeply embedded with philosophical teachings, significantly improves career adaptability by fulfilling basic psychological needs. This traditional Chinese practice emphasizes the balance of yin and yang, encourages focus and concentration, and espouses the principle of using softness to overcome hardness. These elements contribute to career adaptability. The results suggest that Tai Chi can serve as a multifaceted tool for enhancing individuals’ career adaptability, which are crucial in navigating the increasingly complex and dynamic career landscapes faced by today’s college students.

## Data Availability

The raw data supporting the conclusions of this article will be made available by the authors, without undue reservation.
